# Silk Sericin and Its Composite Materials with Antibacterial Properties to Enhance Wound Healing: A Review

**DOI:** 10.3390/biom14060723

**Published:** 2024-06-18

**Authors:** Sheng-Lan Wang, Jia-Jun Zhuo, Shou-Min Fang, Wei Xu, Quan-You Yu

**Affiliations:** 1 College of Life Science, China West Normal University, Nanchong 637002, China; 2023269701j@stu.cqu.edu.cn; 2 School of Life Sciences, Chongqing University, Chongqing 400044, China; 202326131046@stu.cqu.edu.cn (J.-J.Z.); yuqy@cqu.edu.cn (Q.-Y.Y.); 3 Department of Dermatology, Chongqing Hospital of Traditional Chinese Medicine, No. 40 Daomenkou St., District Yuzhong, Chongqing 400011, China

**Keywords:** silk sericin, composite materials, antibacterial, wound healing, mechanism

## Abstract

Wound infections may disrupt the normal wound-healing process. Large amounts of antibiotics are frequently used to prevent pathogenic infections; however, this can lead to resistance development. Biomaterials possessing antimicrobial properties have promising applications for reducing antibiotic usage and promoting wound healing. Silk sericin (SS) has been increasingly explored for skin wound healing applications owing to its excellent biocompatibility and antioxidant, antimicrobial, and ultraviolet-resistant properties. In recent years, SS-based composite biomaterials with a broader antimicrobial spectrum have been extensively investigated and demonstrated favorable efficacy in promoting wound healing. This review summarizes various antimicrobial agents, including metal nanoparticles, natural extracts, and antibiotics, that have been incorporated into SS composites for wound healing and elucidates their mechanisms of action. It has been revealed that SS-based biomaterials can achieve sustained antimicrobial activity by slow-release-loaded antimicrobial agents. The antimicrobial-loaded SS composites may promote wound healing through anti-infection, anti-inflammation, hemostasis, angiogenesis, and collagen deposition. The manufacturing methods, benefits, and limitations of antimicrobial-loaded SS materials are briefly discussed. This review aims to enhance the understanding of new advances and directions in SS-based antimicrobial composites and guide future biomedical research.

## 1. Introduction

Wound infection is a major clinical challenge that significantly hinders wound healing. Anti-infection is a priority in the treatment process, particularly for some chronic wounds, including vascular, diabetic, and pressure ulcers [1]. Previous studies have indicated that most wounds suffer from polymicrobial infections, which are primarily composed of aerobic and anaerobic bacteria and small numbers of fungi and viruses [2,3,4]. Bessa et al. [5] collected 312 wound swab samples from 213 patients with different types of wounds and observed that the most common bacteria were *Staphylococcus aureus* (37%), followed by *Pseudomonas aeruginosa* (17%), *Streptococcus pyogenes* (10%), *Escherichia coli* (6%), and *Corynebacterium* spp. (5%). When a wound is infected by bacteria, increased exudation occurs, and granulation tissue formation at the wound site is inhibited [6]. Wound-infecting bacteria often form a biofilm that allows them to grow and multiply [7]. Massively proliferating pathogenic bacteria usually degrade the extracellular matrix and growth factors [8]. Its metabolites may impair leukocyte function [9] and inhibit keratinocyte migration, thereby reducing re-epithelialization [10]. Moreover, bacterial endotoxins can reduce collagen deposition and induce prolonged elevation of pro-inflammatory cytokines, including IL-1β and TNF-α, which in turn reduce growth factor production [11]. Bacterial exotoxins may also attack many types of cells and cause tissue necrosis [12]. Briefly, inhibition of pathogenic bacterial growth and anti-infection are essential for wound healing.

In traditional treatment, surgical debridement and the appropriate use of antibiotics are the main strategies to prevent wound infection [13,14]. Repeated surgical debridement of unhealed wounds can be traumatic and slower; therefore, more passive localized treatments are often preferred. After the application of topical antimicrobials (chlorhexidine and povidone-iodine), the wound is treated with immobilizing dressings in the form of bandages [15]. Generally, traditional dressings, including cotton, wool, natural or synthetic bandages, and gauze, can be employed as primary or secondary dressings to absorb wound exudates and protect the wound [16]. A previous study suggested that the wound healing rate in a wet environment is faster than that in a dry environment [17]. Conventional dressings do not meet the needs of wound healing in humid environments because bacteria can easily invade soaked dressings. Therefore, ideal dressings retain moisture and exhibit good biocompatibility, mechanical strength, and antimicrobial properties to provide a protective and suitable environment for wound surfaces to promote wound healing and prevent infection [18].

Recently, many biomaterials have been developed to promote wound healing, including silk, alginate, dextran, and hyaluronic acid [19]. Cocoon silk is a natural, moisturizing, and biocompatible material. Silkworm cocoons contain silk fibroin (SF) and silk sericin (SS) layers [20]. Both SF and SS are composed of repetitive amino acid sequences that can form a β-sheet structure. The sequence of SF is [GAGAGS] n, and the partial repeat sequence in SS is GSVSSTGSSSNTDSST [21]. SF also exhibits good mechanical properties, and numerous studies have confirmed the potential of SF antimicrobial composites in promoting wound healing [22]. Although hydrophilic SS does not have good mechanical properties, it exhibits antibacterial, antioxidant, and anti-ultraviolet properties [23,24,25,26]. Moreover, SS can achieve certain mechanical properties by copolymerization or blending with other polymers, including chitosan, polyvinyl alcohol (PVA), alginate, gelatin, collagen, and bacterial cellulose [27,28,29]. Importantly, SS has certain intrinsic antimicrobial properties, which are improved when other antimicrobial agents are added. Various antimicrobial SS composites have been prepared, including hydrogels [30], films [31], sponges [32], and nanoparticles (NPs) [33]. To obtain broad-spectrum antimicrobial properties, inorganic metal NPs, natural antibacterial agents, and antibiotics have been widely explored in the preparation of antibacterial SS biomaterials [34,35,36]. In these composite materials, SS acts as a natural reducing agent for metal NPs and provides a suitable moist environment for wounds and space for the slow release of antimicrobial agents [37]. Therefore, developing antimicrobial SS composite biomaterials and their potential applications in wound treatment has received wide attention.

Many researchers have investigated different strategies for endowing SS composites with a wide range of antimicrobial properties. The combination of SS with different antimicrobial agents has been demonstrated to prevent bacterial infection and accelerate wound healing in skin wounds. However, there has been no systematic review of the methods for preparing these materials and promoting wound healing. In this review, we have discussed antimicrobial agents added to SS composites for skin wound healing, presented their advantages and disadvantages, and outlined recent advances and future trends in this research area. A systematic review of these studies may be beneficial for the future development of antimicrobial SS composite biomaterials.

## 2. The Antibacterial Properties of the Cocoon Silk Sericin Layer

The sericin layer of the silkworm cocoon is predominantly composed of sericin proteins and contains a small number of protease inhibitors, seroins, flavonoids, carbohydrates, salts, and wax [38,39]. At present, five SS genes (*Ser1*, *Ser2*, *Ser3*, *Ser4* and *Ser5*) have been found [40,41,42,43]. Ser1 and Ser3 are the main sericins for silkworm cocoons [43]. Hydrophilic silk sericin has good water solubility, and it also exhibits solution-gel properties [44]. It is easily dissolved into water in the temperature range of 50–60 °C and starts gelling when cooled. Usually, silk sericin solution is obtained from the cocoon by high temperature and high pressure, ethanol precipitation, acidic and alkaline methods [45]. Different extraction methods may affect the molecular weight of sericin and lead to different contents of β-sheet, random coil, α-helix and aggregation chain [46,47,48]. Xue et al. [49] analyzed the growth, integrity, and morphology of bacterial cells after sericin treatment and explored the antibacterial activity against *E. coli* and its interaction mechanism. The antimicrobial activity improved by increasing silk sericin concentration from 10–40 g/L. Sericin proteins can induce blistering in *E. coli* cell membranes. The bacteria then exhibit a decrease in biofilm fluidity and permeability, ultimately changing the media conductivity and outer membrane integrity of *E. coli* cells. Previous studies have revealed that sericin proteins contain a large proportion of serine, glycine, and aspartic acid residues [50,51]. Doakhan et al. suggested that the antibacterial activity of sericin proteins may be due to their cationic nature, which interacts with negatively charged bacterial cell membranes, leading to altered permeability and cell death by inducing protein leakage and other intracellular components of the bacteria [52]. In addition, it has been reported that the antibacterial activity of sericin may be related to the presence of cysteine with sulfhydryl group in its composition [21].

The antimicrobial activity of the SS layer is also attributed to other small components, including protease inhibitors and seroins [39,53]. Protease inhibitors mainly act as antifungal agents. For example, Kunitz-type BmSPI51 [54,55] and TIL-type BmSPI38/BmSPI39 [56,57] can inhibit the growth of *Beauveria bassiana*. Seroins have broad-spectrum antibacterial activity against bacteria, fungi, and viruses [58]. Two types of seroins (seroin 1 and seroin 2) have been identified in the SS layer, both inhibiting the proliferation of nucleopolyhedroviruses [58]. Seroin 2 effectively inhibits the growth of Gram-negative *E. coli* and Gram-positive *Micrococcus luteus*, whereas seroin 1 also inhibits the growth of Gram-positive *Mycoplasma luteus* [58]. Seroins use the same antimicrobial mechanism: binding to bacterial peptidoglycan to inhibit bacterial growth [53]. Moreover, many other small components of the SS layer may exhibit antimicrobial activity. Lysozyme, antimicrobial peptides, lectins, and iron-binding proteins may act specifically as antimicrobial agents [39]. Antimicrobial peptides are the most important components of organisms that resist pathogenic bacteria. Recently, using transgenic technology, an antimicrobial peptide gene has been efficiently overexpressed in the middle silk gland of the silkworm, which may enhance the natural antimicrobial properties of the SS source [59].

## 3. Preparation and Antimicrobial Properties of SS Antimicrobial Composites

**Figure 1:**
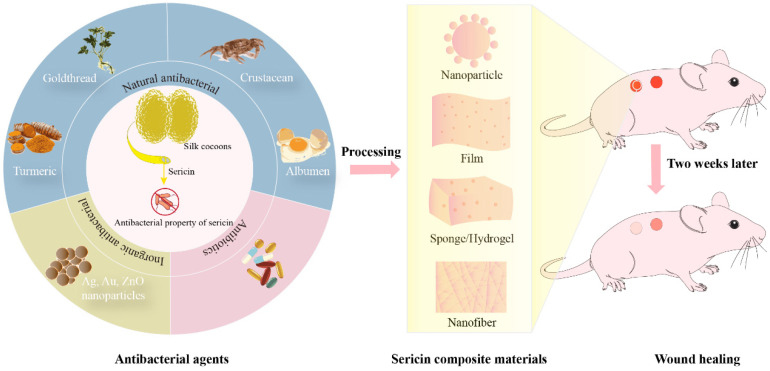
Antibacterial agent-loaded silk sericin composite biomaterials for wound healing. The silk sericin layer isolated from silkworm cocoons has certain intrinsic antimicrobial properties [39,49]. Antimicrobial agents added to SS composites fall into three main categories, inorganic antimicrobials, natural antimicrobials, and antibiotics. SS-based antimicrobial composites can be processed and prepared into different material types including nanoparticles, films, sponges, hydrogels and nanofibers [30,32,33,62,63]. The SS composites accelerated wound healing in mice compared to controls [30,64,65].

Although the SS layer of the silkworm cocoon has antimicrobial properties, it mainly resists microorganisms in the environment where silkworms live. These microorganisms may differ somewhat from the pathogens in human wounds. Therefore, the antimicrobial properties of SS alone are insufficient to achieve the purpose of completely inhibiting the growth of pathogens in human wounds. Other antimicrobial components are also necessary for the preparation of antimicrobial composites and functional materials. Recently, antimicrobial SS composite materials have been prepared by loading inorganic metal NPs, natural antibacterial agents, and antibiotics (Figure 1) [33,34,60,61]. Numerous studies have indicated that SS composite materials exhibit broad-spectrum antimicrobial properties and can promote wound healing, showing promising applications (Table 1).

### 3.1. SS Nanoparticle Composite Materials and Antimicrobial Properties

Metal NPs are known to exhibit broad-spectrum antimicrobial properties [84]. The antibacterial mechanism of NPs is that they can inhibit bacteria by inducing oxidative stress [85,86] and releasing metal ions [87]. Metal NPs slowly release metal ions that can cross membranes and disrupt cellular processes inside the cell [88]. Chemical reduction is the most versatile, economical, and easiest method for controlling the shape and size of metal NPs. However, the preparation of metal NPs usually requires the addition of toxic chemicals that are unfavorable for skin wound treatment. Toxic chemicals, including sodium borohydride, hydrazine, aniline, cetyltrimethylammonium bromide, and sodium dodecyl sulfate, have been used as reducing and stabilizing agents for the preparation of metal NPs [89]. Previous studies have revealed that natural SS act as reducing (the carboxylic acid group) and stabilizing agents for metal NPs and are environmentally friendly [74,90]. Silver (Ag), gold (Au), and zinc (Zn) nanoparticles have been widely used to prepare antibacterial SS composite materials [30,31,75]. The SS materials loaded with metal NPs effectively inhibit the skin-infecting bacteria, including *E. coli*, *P. aeruginosa* and *S. aureus* [30,32,66,68,91]. The shape, charge, size, high specific surface area, and high reactivity of NPs (1–100 nm in diameter) render them ideal antimicrobial weapons [92]. The use of NPs offers a prospective approach for controlling infections caused by multidrug-resistant bacteria [93]. This process of synthesizing NPs is simple and environmentally friendly compared to the traditional, tedious, and costly physical and chemical methods containing hazardous chemicals [36].

#### 3.1.1. SS Composites Loaded with Ag Nanoparticles

Silk sericin is a natural biomaterial exhibiting excellent biocompatibility and biodegradability, promoting cell adhesion and proliferation [94]. Ag nanoparticles (AgNPs) have been shown to inhibit the growth and survival of bacteria, including human and animal pathogenic bacteria and fungi [95]. AgNPs containing silver atoms can be prepared by reducing silver nitrate [96]. The Ag atoms in AgNPs are first oxidized and then released as Ag ions [97]. The surfaces of smaller AgNPs contain more atoms at the corners and in the lattice interstices, resulting in a more reactive surface and higher release. A favorable compatibility between AgNPs and SS has been identified, allowing AgNPs to be safely embedded in SS materials [68,74]. Carboxylic acid groups in SS have been proposed to function as reducing agents during the synthesis of AgNPs, whereas NH^2+^ and COO^−^ serve as stabilizers for AgNPs [91]. Therefore, in situ preparation of SS-AgNPs composites in SS and AgNO_3_ solutions does not require any reducing agents or expensive instrumentation [74]. A comparison of the antimicrobial activity of SS-AgNPs with that of AgNO_3_ (3 mM) showed that the inhibition zone of SS-AgNPs against drug-resistant *E. coli* and *P. aeruginosa* was greater than that of AgNO_3_ solution [91]. Moreover, SS is highly stable over a wide range of temperatures and pH values [33]. Therefore, the use of highly stabilized SS as a reducing agent for the synthesized AgNPs can greatly extend their lifetime.

The SS composite system for the in situ synthesis of AgNPs contains several materials that serve other purposes (providing mechanical strength) in addition to the reducing agent SS [37,64,66,69,71,72]. PVA solution (2%, *w*/*v*, 5 mL) and different concentrations (0.1, 0.2, and 0.5 mM/L) of AgNO_3_ were added to SS solution (2%, *w*/*v*, 5 mL) under continuous stirring. The mixture was placed in a petri dish under natural light at 25 °C for 24 h to promote the synthesis of AgNPs. Finally, the mixture was repeatedly freeze–thawed and lyophilized to prepare the AgNP-SS/PVA dressing [37]. SS/PVA dressings had no inhibitory effect on bacterial growth, whereas SS/PVA-AgNPs dressings showed antagonistic effects against *E. coli*, *P. aeruginosa* and *S. aureus* [37]. Recently, an SS-methacryloyl/Ag cryogel (SMC@Ag) has been shown to exhibit better hemostatic and antimicrobial properties than commercial gelatin sponges without a significant inflammatory response [66]. The purified SS powder and methacrylic anhydride (MA) were stirred in phosphate-buffered saline and dialyzed. After lyophilization, the SS-MA powder was obtained. Then, an ammonium persulfate aqueous solution and TEMED were added to the pre-cooled SMA aqueous solution at 4 °C. After storage at −20 °C for 15 h, the mixture was left at room temperature for 30 min and repeated to obtain the SS-MA cryogel (SMC). Subsequently, SMC@Ag cryogels were generated by immersing the SMC in different concentrations of AgNO_3_ aqueous solution. A significant growth inhibition band was present in SMC@Ag compared with SMC, which exhibited potent antimicrobial properties against *S. aureus*, *MRSA*, and *E. coli* [66]. Furthermore, the release of Ag from SS composite dressings included a burst phase and a steady state phase [37]. The burst phase was attributed to the diffusion of loosely bound Ag, and the steady-state phase indicated sustained release of Ag. This suggests that SS composite dressings containing AgNPs had sustained antimicrobial activity [37,71]. Notably, when the concentration of SS-AgNPs complex was increased to 25 mg/L, the morphology of some 3T3 cells was altered, suggesting that this concentration is toxic to 3T3 cells [74].

#### 3.1.2. SS-ZnONPs Composite Materials

ZnO nanoparticles (ZnONPs) exhibit antimicrobial properties due to increased specific surface area [98]. Direct contact of ZnONPs with the cell wall leads to the disruption of bacterial cell integrity, the release of antimicrobial ions (Zn^2+^), and the formation of reactive oxygen species (ROS) [98]. These ROS, including O^2−^, OH^−^, and H_2_O_2_, are very effective in destroying the bacterial respiratory chain and DNA and protein functions, leading to cell death [99]. Another antibacterial mechanism of ZnONPs is the release of Zn^2+^, which can inhibit the active transport of the membrane and destroy the enzyme system and amino acid metabolism [100,101].

The SS/PVA films were prepared by blending SS with PVA, and the composite films were immersed in polydopamine (PDA) solution, and then the PDA-SS/PVA films were immersed in ZnONPs solution to obtain ZnONPs-PDA-SS/PVA composite films [31]. The number of colonies was much lower in the presence of the ZnONPs-PDA-SS/PVA films than in the control. Similarly, ZnONPs could be loaded into an SS/PVA sponge, and the addition of ZnONPs improved the antibacterial properties of the SS/PVA sponge. ZnONPs-SP sponge had a significant inhibitory effect on the growth of *E. coli* and *S. aureus*, which was higher than that of SS and SS/PVA groups [32]. Cell viability assays confirmed that after 48 h, cell viability in the presence of ZnONPs-SP sponges did not differ from that in the presence of control, SS, and SP sponges [32]. Furthermore, ZnONPs-SP sponges showed excellent cytocompatibility for NIH3T3 cells without affecting cell growth.

#### 3.1.3. SS-AuNP Composite Materials

Gold nanoparticles (AuNPs) have high relative inertness and biocompatibility [102,103]. The antimicrobial effect of AuNPs may be due to their small size and accumulation at specific locations in the bacterial cell, leading to membrane rupture and lysis [104]. Several hydroxypolar amino acids (serine and threonine) of SS can be used as reducing agents to prepare SS-AuNPs [90]. The SS solution was added dropwise to a conical flask containing gold (III) chloride solution (HAuCl_4_·3H_2_O) and then irradiated with a UV lamp at room temperature for approximately 24 h [36]. Synthesis of SS gold nanoparticles (SSP-AuNPs) was completed when the color of the reaction mixture varied from transparent to purple-red-violet. The results indicated that when the concentration of SSP-AuNPs reached 100 μg/disc, it was very effective against four bacteria, including *Escherichia coli*, *E. faecalis*, *Salmonella enterica*, and *S. typhimurium*, with the diameter range of inhibition zone from 11.10–14.96 mm.

#### 3.1.4. SS Composite Materials Loaded with Multiple Metal Nanoparticles

Recent studies have revealed that bimetallic nanoparticles that combine the properties of two metals in a single nanostructured system exhibit promising applications [105]. For example, ZnONPs are well-dispersed and can effectively prevent the aggregation of AgNPs, and AgNPs/ZnONPs hybrids have a synergistic antimicrobial effect that can inhibit bacterial growth or kill bacteria [106,107]. Das et al. [105] used SS as a reducing agent to synthesize bimetallic gold-silver nanoparticles (Au/AgNPs), called BM-GS NPs. In addition to being a reducing agent, SS can also be used as a capping agent and stabilizer for bimetallic gold-silver nanoparticles. Compared with single-metal nanoparticles, bimetallic nanoparticles exhibit higher durability due to the combined advantages of the two metal components. Li et al. [76] prepared a composite film (MPAZ) loaded with ZnONPs and AgNPs by coating PDA on the surface of the SS/agarose film. The addition of ZnO to AgNPs is an effective method for reducing the potential cytotoxicity of AgNPs, and it also has a synergistic antimicrobial effect in inhibiting bacterial growth. The MPAZ showed a significant reduction in the number of colonies for both *E. coli* and *S. aureus* compared to MP (SS/AG), MPZ (ZnO-PDA-SS/AG), and MPA (AgNPs-PDA-SS/AG).

In summary, at appropriate concentrations, metal nanoparticles have low toxicity to human cells and are biocompatible. Importantly, SS exhibits good slow-release properties on metal nanoparticles and achieves long-lasting antimicrobial effects [37,68]. Moreover, they can release antibacterial ions for a long time, thus maintaining the antibacterial impact over an extended period and reducing the risk of bacterial reinfection. However, when metal nanoparticles are used, any potential risk to human toxicological events is attributed to their physicochemical properties, dosage, and route of administration [103]. Several studies have demonstrated the cytotoxic and genotoxic effects of AgNPs on several cell types, including fibroblasts and keratinized cells [108,109]. Therefore, AgNPs should be monitored for plasma levels of silver when applied as a dressing to avoid adverse systemic effects.

### 3.2. SS Materials for Slow Release of Drugs

Antibiotic drugs are often used to treat wounds and prevent infections. However, the usual treatment dosage is high and has a short duration of efficacy, causing resistance in pathogenic bacteria. The slow release of drugs may reduce the usage amount and increase the duration. Due to its amphiphilic nature (polar side chains and hydrophobic structural domains), SS is expected to form hydrophobic matrices and bind charged molecules through its polar side chains. This interaction can also facilitate the sustained release of certain drugs [110]. Tao et al. [28] evaluated the potential of SS/PVA hydrogels as drug delivery vehicles using gentamicin and aspirin as model drugs. Since SS contains many positively (His, Lys, and Arg) and negatively (Asp and Glu) charged amino acid residues, gentamicin and aspirin are basic and acidic compounds, respectively. Electrostatic interactions between opposite charges favored the interaction of the drugs with the SS/PVA hydrogels. Moreover, the specific hydrophilic/hydrophobic interactions between the drug and SS/PVA hydrogel can also enhance the interaction of the drug with the SS/PVA hydrogel. Gentamicin and aspirin showed a 10 and 20 h burst release, respectively, while the steady-state phase showed a slow release of activity. Therefore, SS can immobilize drugs in its structure through an ion exchange mechanism. This exchange not only facilitates drug loading but also slows down the release rate of the drug to achieve a slow-release effect [111].

Copolymerization, cross-linking, or blending SS with other substances is a commonly used method to form various scaffolding materials for better performance, due to its biodegradability, easy availability, hydrophilicity, and carrying a variety of polar side groups [34,112,113], e.g., to improve their mechanical properties and overcome their natural fragility [28,70,114]. Also, this process may involve covalent bonding, hydrogen bonding, ionic bonding, or hydrophobic interactions between the SS and other additives. This also impedes the free diffusion or release of the drug, thus achieving a slow-release effect. Bakadia et al. [34] investigated the potential of BC-PVA@SSga loaded with azithromycin in treating wound infections. Azithromycin (AZM) is the first macrolide antibiotic among aza-drugs and is also an FDA-certified antibacterial drug [115]. The prolonged linear release of AZM for more than 35 h indicates that it is a sustained drug release. This sustained release may be because protein genipin, which is a natural cross-linking compound, contains reactive groups (-OH), while SS contains a large number of polar groups (-NH_2_) with which it cross-links to form a rigid matrix, and the crosslinked sericin obstruct the discharge of curcumin into the dissolution medium [116].

Furthermore, embedding drug-encapsulated microspheres or nanofiber particles into a hydrogel matrix enhances the control of slow drug release compared with a single carrier [117]. Bakadia et al. loaded vancomycin (VA) and gentamicin (GEN) into PVA microspheres and co-embedded them in PVA/SS hydrogels to study the effect on burn wound healing [80]. Compared with hydrogels without microspheres, the microsphere hydrogels had a longer uptake time and sustained drug release without a significant rupture effect. After seven weeks, the microsphere hydrogels all showed a degradation rate of approximately 70%, suggesting that biologically active molecules can be released sustainably while still providing sufficient time for burn wounds to heal completely. Diab et al. [83] optimized the ability of the prepared SS/propolis nanoparticles to effectively release the drug load (amoxicillin) by using different SS concentrations and stirring times so that the prepared nanoformula can gradually release the drug load. In the initial release, the prepared nanoformulations showed a significantly delayed-release rate (*p* < 0.05). After 2 h, the cumulative release of amoxicillin reached 5%, and sustained release occurred due to drug diffusion (amoxicillin) from the nanoformula. Encapsulation of drugs by SS/propolis nanoparticles enables long-lasting release of active drugs, thus overcoming the problem of short half-life of drugs due to rapid cumulative release.

### 3.3. SS Materials Loaded with Natural Antimicrobial Products

Natural antimicrobial products are abundant and readily available through extraction and are primarily classified as plant and animal extracts. Compared to inorganic metal nanoparticle antimicrobials and antibiotics, natural antimicrobial products generally exhibit lower toxicity and fewer side effects. The preparation of SS composites utilizing the antimicrobial properties of natural products has received considerable attention in recent years.

#### 3.3.1. SS Composites Loaded with Plant Extracts

Curcumin, a natural pigment extracted from turmeric, is an acidic polyphenolic compound with anti-inflammatory and antibacterial effects against *S. aureus* [118]. Cur-SNPs were prepared by adding ethanol containing curcumin (10 mg) to the SS solution and cross-linking it with genipin [116]. A higher SS content (4% *w*/*v*) resulted in larger particle formation. The encapsulation efficiency and curcumin loading of Cur-SNPs were 85.7% ± 1.20% and 47% ± 1.23%, respectively. At pH 7.4, Cur-SNPs showed 14.1% ± 1.5% and 46.6% ± 0.94% curcumin release at 2 and 24 h, respectively. Sustained release was observed within 60 h, and the sustained release rate reached 83.1% ± 1.0%. Sustained release may be due to the cross-linking of SS with genipin, leading to rigid protein matrix formation. Cross-linked SS impedes the release of curcumin into the culture medium. Additionally, Chuysinuan et al. [60] reported that the CMC/SS dressing, as an effective drug delivery device, can release curcumin from the dressing in a controlled-release manner within 48 h, with an initial burst release rate of up to 40% after the first hour and a gradual release rate of 60–80% after 24 h.

Demethoxycurcumin (DMC) and bisdemethoxycurcumin (BDMC) are natural analogs of curcumin that lack one or two methoxyl groups on the benzene ring of the parent structure, providing better stability and outstanding anti-inflammatory and antiviral properties [119,120]. However, their low water solubility and stability lead to poor bioavailability and limited clinical efficacy. Singh et al. [62] developed a vesicle-encapsulated DMC or BDMC and loaded it onto a biodegradable PVA/gelatin/SS film. Specifically, vesicles with or without DMC/BDMC suspensions were prepared using film hydration, ultrasound, and extrusion. Composite membranes were then prepared using a blend of PVA, gelatin, SS, and vesicles cast on a mold and dried at room temperature. This vesicle-based drug delivery (VDD) system is a key technology for addressing the challenges of drug solubility, permeability, bioavailability, and intracellular uptake [121]. The results displayed that vesicle-embedded DMC exhibited enhanced antimicrobial activity against *A. baumannii* and *S. epidermidis* compared to unembedded DMC [62].

Berberine is an isoquinoline derivative with anti-inflammatory and antimicrobial properties, which has been used for treating wound healing when embedded in vesicle carriers, gel delivery systems, and cream formulations [122,123,124]. This method of encapsulating drugs into vesicles and encapsulating vesicles into semi-solid formulations (hydrogels) has been confirmed to be an important strategy to circumvent the drawbacks of nanovesicles, and the sequestration of such systems in polymer matrices provides a means of further controlling the spatial and temporal release of drugs [125,126]. Yan et al. [35] prepared berberine-loaded SS and poly(ethylene glycol) diacrylate (SS/PEGDA) hydrogels by freeze-drying. With an increase in SS content, the porosity of the scaffold structure changed from macropores to mesopores. Continuous addition of SS led to a higher cross-linking density, which improved the compressive strength of the SS/PEGDA scaffolds. The 20% SS/PEGDA scaffold exhibited the largest specific surface area and appropriate mechanical properties. The antimicrobial properties of berberine-loaded SS/PEGDA hydrogels were evaluated by the zone of inhibition, which indicated that the antimicrobial agent released from SS/PEDDA hydrogels prevented the formation of bacterial colonies and that *S. aureus* was more susceptible to berberine than *E. coli* [35].

#### 3.3.2. Composites of SS with Animal Extracts

Chitosan, a polysaccharide derived from chitin and a biopolymer extracted from animal shells, is frequently used as a biomaterial in wound dressings due to its biocompatibility, degradability, and broad-spectrum antibacterial properties [118]. The antibacterial properties of chitosan are because the contact between positively charged chitosan and negatively charged bacterial surface molecules alters the permeability of the bacterial membrane, leading to leakage of internal components and cell death [127]. Chitosan mixed with SS can prepare hydrogels [77], nanofibers [78], scaffolds [128], and other materials for wound healing. Cross-linking of SS with chitosan enhances thermal stability and promotes cell attachment and viability of human dermal fibroblasts while reducing inflammation and hemolysis [77]. Hydrogels were prepared using different concentrations of genipin, optimized chitosan, and different ratios of SS, and the antimicrobial properties of the hydrogels were evaluated using Gram-negative (*E. coli*) and Gram-positive (*S. aureus*) bacteria by agar diffusion method [77]. The results showed that the zone of inhibition of the hydrogel against *E. coli* was estimated to be between 8 and 12 mm, and that the inhibition was optimal when the ratio of CS to SS was 1:2. The zone of inhibition for *S. aureus* was 10–15 mm.

Lysozyme has been widely used as an antimicrobial agent in wound dressings [79,129,130]. The antimicrobial activity of lysozyme is attributed to its ability to hydrolyze the β-1, 4 linkage between N-acetylmuramic acid and N-acetyl-glucosamine in the peptidoglycan of the microbial cell wall [131]. Egg white is one of the rich sources of lysozyme [132]. In a previous study, an SS/agarose gel was immersed in a lysozyme solution, removed, and freeze-dried to obtain an SS/AR/LZM gel [79]. Because SS is negatively charged and lysozyme is positively charged, the adsorption of lysozyme onto the SS/AR gel can be attributed to the electrostatic interaction between the opposite charges of SS and lysozyme. Moreover, the special hydrophilic/hydrophobic interactions between lysozyme and SS can enhance the adsorption of lysozyme. These results indicate that lysozyme release can be divided into burst and stabilization phases. During the initial burst phase, the release rate was relatively high. This effect is related to the diffusion of water molecules and desorption of lysozyme near the gel surface. During the stabilization phase, lysozyme was gradually released from the SS/AR/LZM gel for up to 60 h at a 99% release rate. Lysozyme released from the SS/AR/LZM gel inhibited the growth of both *E. coli* and *S. aureus*. The bacterial inhibition of *E. coli* and *S. aureus* by S50A50L20 and S50A50L50 gels was 76%/87% and 84%/95%, respectively. Additionally, the S50A50L75 gel completely inhibited the growth of *E. coli* and *S. aureus*, suggesting that increasing the concentration of lysozyme improved the antimicrobial capacity of the composite gel because of the increased loading and release of lysozyme.

Natural antibacterial products have the advantages of being a wide source, having low toxicity, and biocompatibility, which may provide a choice for improving antibacterial materials and solving the problem of antibiotic resistance [133]. However, the application of natural antimicrobial agents is limited by their inherent properties, such as curcumin’s instability and aqueous insolubility [134]. Notably, when natural antibacterial products were added to SS composites, a slow release effect can be achieved due to electrostatic interactions, hydrophilic/hydrophobic interactions, and special material types (vesicles) [35,60,79]. Therefore, developing SS materials containing natural antimicrobial products requires further investigation.

## 4. SS-Based Composite Material Promotes Wound Healing

### 4.1. Wound Healing Stages

Normal wound healing occurs in four major stages: hemostasis, inflammation, proliferation, and remodeling [135]. Exposure of blood to subendothelial tissue activates protein hydrolysis cleavage and coagulation cascade reactions during the hemostatic phase. Platelets aggregate to form hemostatic clots and release growth factors, including platelet-derived growth factor, transforming growth factor-β, epidermal growth factor (EGF), and insulin-like growth factor, thereby triggering angiogenesis and activation of fibroblasts, endothelial cells, macrophages, and neutrophils. During the inflammatory phase, neutrophils and macrophages play a role in removing bacteria, damaged tissue, and foreign materials, thereby preventing infection. Macrophage activation leads to the release of growth factors, which triggers subsequent cellular responses. During the proliferative phase, fibroblasts migrate and deposit the extracellular tissue matrix, including fibrous proteins, collagen, polysaccharides, proteoglycans, glycosaminoglycans, and fibronectin, which appear as granulation tissue. Wound contraction occurs as myofibroblasts interact with the extracellular matrix (ECM) through connective tissue contraction, effectively bringing the wound edges closer together. Finally, a remodeling phase occurred. Therefore, the healing process cannot occur without the involvement of fibroblasts, keratin-forming cells, proteins, hormones, cytokines, and enzymes [136].

**Figure 2:**
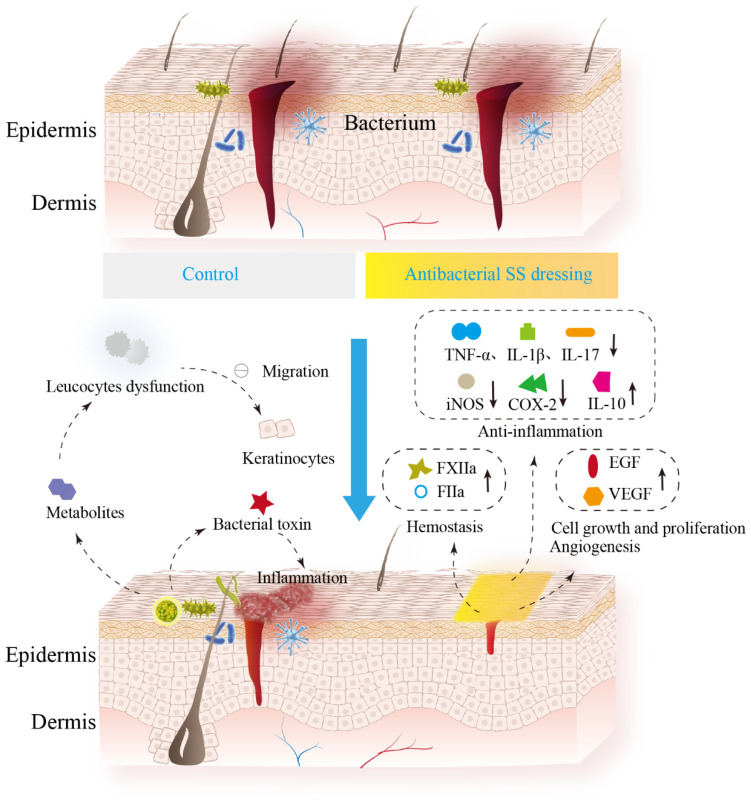
Mechanism of skin wound healing promoted by SS-based antimicrobial composites. The skin contains the epidermis and the dermis [140]. In the control group, toxins produced by bacteria elevate pro-inflammatory cytokines (IL-1β, TNF-α) and increase the inflammatory response in wounds [11]. Bacterial metabolites can also cause leukocyte dysfunction [9], which inhibits migration of epidermal cells and keratinocytes [10]. For the treated group with SS-based antimicrobial dressing, pathogenic bacteria proliferation was significantly inhibited and it can promote wound healing through hemostasis, anti-inflammation, cell proliferation and angiogenesis [34,66,67,80,141,142,143]. In particular, SS-based antimicrobial materials are favorable for hemostasis through increasing the activity of FXIIa (the initiator of the coagulation cascade) and FIIa (the final thrombin in the coagulation cascade) [66]. The dressings also has anti-inflammatory properties through down-regulating pro-inflammatory cytokines such as TNF-α, IL-1β, and IL-17 [144] as well as synthase enzymes involved in the inflammatory process, including COX-2 (cyclooxygenase) and iNOS (inducible nitric oxide synthase) [142] and increasing the anti-inflammatory cytokine IL-10 [141]. Inhibition of pathogenic bacteria will increase growth factor secretion and further promote cell proliferation, migration and angiogenesis [34,143].

There is often a symbiotic relationship between bacteria and host immune factors that do not cause an inflammatory response in healthy skin [137]. However, the presence of skin wounds can disrupt this dynamic balance and lead to the colonization and multiplication of pathogens, which can cause infection and inflammation to delay wound healing (Figure 2) [138]. Developing wound infection depends on the toxins released by bacteria and the intensity of the host response. In infected acute wounds, the normal healing process is disrupted, leading to poor healing and potential chronic wound development [137]. Many species of bacteria produce toxins and other metabolites that inhibit the migration of the skin epithelium and digest the tissue proteins and polysaccharides present in the dermis. Bacterial endotoxins cause elevation of pro-inflammatory cytokines, including IL-1β and TNF-α [11]. Bacterial exotoxins can attack many cell types and cause tissue necrosis [12]. Bacteria present in acute and chronic wounds live in colonies encased in an autocrine extracellular polysaccharide (EPS) matrix known as a biofilm and are less susceptible to the innate immune system and therapeutic antibiotics [139]. Due to the mechanical barrier formed by the EPS and the bacterial products in the matrix, it is also difficult for phagocytes to penetrate the biofilm, thus reducing bacterial phagocytosis by phagocytes.

Anti-infection is a crucial task in the wound-healing process, especially for some chronic wounds. To enhance the antimicrobial properties of SS materials, different antimicrobial agents, including metal nanoparticles, natural antimicrobials, and antibiotics, have been incorporated into the composites, which have significantly promoted wound healing [30,34,37,64,66,75,81,82]. Moreover, some studies have demonstrated the anti-inflammatory effects of SS alone [142]. Furthermore, SS composites exhibit better hemostatic properties (Figure 2) [66]. A previous study showed that SS-methacryloyl/silver cold gel (SMC@Ag) exhibits rapid hemosorption and procoagulant activity [66]. The condensation activation mechanism may be due to the negative charge and high roughness of the SMC@Ag surface, which induces activation of the condensation pathway. Furthermore, SS facilitates platelet adhesion, which promotes coagulation [66]. Therefore, SS composites loaded with antimicrobial agents may be involved in multiple wound-healing processes, including hemostasis, anti-inflammation, and anti-infection.

### 4.2. SS Composites Loaded with Metal Nanoparticles Promote Wound Healing

SS composites loaded with various metal nanoparticles can promote wound healing by inhibiting common chronic wound bacteria [30,37,75]. Sodium alginate, different concentrations of AgNO_3_, and calcium gluconate were sequentially added to SS, mixed thoroughly, and placed under natural light for 24 h at 25 °C to obtain SA/Se-Ag hydrogels [30]. It was suggested that SA/Se-Ag0.2 (with 0.2 mmol/L AgNO_3_) promotes wound healing more effectively than SA/Se and control [30]. On day 12, wounds treated with SA/Se-Ag0.2 were almost completely healed, while wounds in the SA/Se and control groups were still visible. The SA/Se-Ag0.2 group showed a significant reduction in the number of colonies and inflammatory cells, indicating that antimicrobial activity is essential for wound healing. Furthermore, the quality of wound angiogenesis recovery was significantly improved after treatment with SA/Se-Ag0.2. Similar results were reported in other studies [37]. Consequently, in addition to antimicrobial and anti-inflammatory functions, SS dressings loaded with AgNPs might be involved in angiogenesis and collagen deposition, which may promote wound healing.

### 4.3. SS Composites Loaded with Natural Antimicrobial Products Promote Wound Healing and Mechanism

Studies have confirmed that SS composites loaded with plant extracts have potential applications in promoting wound healing [141]. After comparing several plant extracts, the results indicated that the SS composite hydrogel containing ripe banana peel extract exhibited the best anti-inflammatory effect [141]. Mice treated with the SS + banana peel extract hydrogel showed a significant increase in pro-inflammatory cytokine (IL-6) levels on day 5 after surgery and a decrease on day 11. Meanwhile, the IL-6 levels in the negative control group increased on day 11 after surgery. After wound induction, IL-10 levels significantly increased in mice treated with the SS + banana peel extract hydrogel. There was a significant difference in the percentage of wound contraction in the SS + banana peel extract and SS + curcumin treatment groups on day nine as compared to the positive control (Polyfax). These results suggest that SS composites loaded with plant extracts promote wound healing, mainly through antimicrobial action and modulation of the inflammatory response (Figure 2) [141].

Lupeol can also be added to SS complexes along with other antimicrobial and anti-inflammatory reagents including chitosan and AgNPs to accelerate wound healing. Lupinol is a plant extract that promotes the formation of new blood vessels [145]. Chu et al. [64] prepared a novel temperature-sensitive self-assembled SS hydrogel loaded with Ag-modified CS nanoparticles and lupeol (CS-Ag-L-NPs). The wounds in the CS-L-NPs and CS-Ag-L-NPs groups were essentially healed by the 21st day compared to the other 3 groups without lupinol, demonstrating that nanoparticles loaded with lupinol accelerated wound healing [64]. For the CS-Ag-L-NPs gel group, wound re-epithelialization was faster than other groups, and the wounds were completely re-epithelialized and appendages and hair follicles appeared on day 21. Further studies indicated that the lupeol could activate PI3K/Akt and p38/ERK/MAPK pathways to promote wound healing [146].

### 4.4. SS Composites Loaded with Antibiotics Promote Wound Healing

The addition of various antibiotics to SS composite materials has been confirmed effective in accelerating wound healing [34,65,80,81,82]. Tigecycline exhibits potent in vitro activity against most Gram-positive and Gram-negative aerobic and anaerobic bacteria [147]. Tigecycline-loaded SS composites (TC-SS/PVA) showed sustained tigecycline release [82]. Wound sizes treated with TC-SS/PVA decreased by about 99% compared with approximately 80% in the blank control. In another study, a cellulose/poly(vinyl alcohol)@SS/AZM scaffold called BC-PVA@SSga loaded with AZM showed favorable therapeutic effects on wounds in mice [34]. On day five, wounds in the BC-PVA@SSga treatment group were as clean as those in the control group (uninfected wounds). Furthermore, wound healing was higher in the SS-containing BC-PVA@SSga group than that in the 0.9% NaCl and control groups. Meanwhile, the myeloperoxidase activity of the inflammatory marker content was lower in the BC-PVA@SSga group than in the control and NaCl groups. In the control and NaCl groups, the prolonged inflammatory response persisted until day 14, which was primarily associated with the presence of *S. aureus* in the wound. In promoting the wound healing process, elevated expressions of VEGF and CD31 were observed in BC-PVA@SSga, indicating neovascularization on day seven. Furthermore, CK14 expression in the BC-PVA@SSga group on day 14 highlighted complete re-epithelialization [34]. Comparable results have been achieved with alternative SS composites containing antibiotics, which facilitate wound healing primarily by reducing inflammation and promoting angiogenesis and re-epithelialization (Figure 2) [65,80,81]. To explore the relationship between prolonged retardation and promotion of wound healing, embedding vancomycin (VA) and gentamicin (GEN)-encapsulated microspheres in SS hydrogels enhanced the control of sustained drug release [80]. Microsphere hydrogels containing VA and GEN provide enhanced antimicrobial activity, higher water absorption, and faster burn wound healing than drug-free microsphere hydrogels and commercial Tegaderm™ films.

## 5. Conclusions and Future Trends

SS has received increasing attention in the biomedical field. SS-based materials can achieve certain mechanical properties by copolymerization or blending with other polymers, including chitosan, PVA, alginate, gelatin, collagen, and bacterial cellulose. Different types of antimicrobial agents (metal nanoparticles, natural antimicrobials, and antibiotics) have been widely added to SS-based materials to enhance their antimicrobial and wound-healing potential. Among these, SS can be used as a reducing agent (the carboxylic acid group) for preparing metal nanoparticles, a process that is environmentally friendly and free of toxic chemicals. Moreover, SS enables the slow release of antimicrobial agents, thereby minimizing overexposure, reducing the number of dressing changes, and alleviating patient pain. Although much research has been conducted in this field, the different advantages and disadvantages of various antibacterial agents limit the wider use of SS composites loaded with antibacterial agents. For example, owing to the small size of metal nanomaterials, intracellular aggregation is possible, which is believed to be an important cause of their cytotoxicity [148]. They may also cause dose-dependent toxicity in mammalian cells, including oxidative stress and DNA damage, ultimately leading to cell death. In summary, the SS composites with appropriate concentrations of antimicrobial agents such as metal nanoparticles exhibited non-toxicity or very low cytotoxicity, good biocompatibility, and good healing results in mouse experiments [30,37,67]. This provides a good practical basis for further application in practice. Natural antibacterial products have limited antibacterial effects and poor heat resistance, whereas antibiotics may cause drug resistance. Therefore, the advantages and disadvantages should be assessed when selecting antibacterial agents. Designing combinations of different types of antimicrobial agents may be a potentially beneficial approach for reducing the dosage of certain agents. However, better results can be achieved through synergistic action of multiple antimicrobial agents.

Live bacterial therapy treats various inflammatory and immunopathological disorders through bacterial interference and immunomodulation and has received much attention [149]. Some beneficial bacteria secrete large amounts of metabolites and antimicrobial drugs to create unique localized microenvironments and inhibit the growth of competing microorganisms [150,151]. Beneficial bacteria have been widely explored for diagnostic and therapeutic purposes [152,153]. Recently, Ming et al. [154] prepared novel hydrogel scaffolds containing live bacteria encapsulated in hydrogel microspheres with excellent anti-infective and accelerated wound healing capabilities. In vitro experiments showed that the encapsulated bacteria did not escape into the environment and that the hydrogels exhibited good resistance to pathogenic bacteria. Moreover, live-bacteria-hydrogel-treated mice showed less inflammation and faster healing at the wound site. This strategy can open a new window for live bacterial therapy as a safer treatment and promote the use of bacteria in treating various diseases. Silk sericin composites are expected to improve wound repair if combined with other bioactive substances, including beneficial microorganisms.

Finally, the study of antimicrobial mechanisms and the development of slow-release materials are particularly important for enhancing the antimicrobial effect and prolonging the duration of antimicrobial persistence, which can lay a solid foundation for clinical use. Therefore, new antimicrobial compositions and mechanisms need to be continuously explored to find more effective and long-lasting antimicrobial methods. It is also possible to identify crucial inhibitory factors for bacterial growth by studying the processes of bacterial growth and reproduction. This may interfere with bacterial metabolism, destroy the cell wall structure, and block bacterial DNA or protein synthesis to achieve antimicrobial purposes. In conclusion, SS composites loaded with antimicrobial agents offer a promising strategy for promoting wound healing. Future research and development can further refine the properties and applications of these materials, contributing to the realization of more effective, safe, and sustainable wound healing protocols.

## Figures and Tables

**Table 1:** Antimicrobial spectrum and efficiency of SS-based antimicrobial composite biomaterials for wound healing.

Categorization		Reagent Content	Forms	Antimicrobial Testing	Research Model	Efficiency	References
Metal nanoparticles	Ag	0.8 mmol/L AgNO_3_	hydrogel	*E. coli*, *S. aureus*, *P. aeruginosa*	In vitro, rat model	antibacterial, anti-inflammatory, promoting angiogenesis	[30]
		0.01 M AgNO_3_	hydrogel	*S. aureus*, *MRSA*, *E. coli*	In vitro, rat model	antibacterial, hemostasis	[66]
		0.05 wt% AgNO_3_	hydrogel	*E. coli*, *S. aureus*	In vitro, rat model	antibacterial, promoting cell migration and proliferation, hemostasis	[67]
		10 mM AgNO_3_	film	*E. coli*, *S. aureus*	In vitro	antibacterial, promoting cell proliferation	[68]
		50 mM AgNO_3_	film	*E. coli*, *S. aureus*	In vitro	antibacterial	[69]
		30 mM AgNO_3_	film	*E. coli*, *S. aureus*	In vitro	antibacterial	[70]
		20 mM AgNO_3_	film	*E. coli*, *S. aureus*	In vitro	antibacterial	[71]
		100 mM AgNO_3_	film	*E. coli*, *S. aureus*	In vitro	antibacterial	[72]
		0.2 mM/L AgNO_3_	sponge	*E. coli*, *S. aureus*, *P. aeruginosa*	In vitro, rat model	antibacterial, anti-inflammatory, promoting wound healing	[37]
		5 mM AgNO_3_	sponge	*E. coli*, *S. aureus*	In vitro	antibacterial	[73]
		30 mg AgNO_3_	nanoparticle	*E. coli*, *S. aureus*, *K. pneumoniae*	In vitro	antibacterial	[33]
		4 mg/mL AgNO_3_	\	*S. aureus*	In vitro	antibacterial	[74]
	Au	1.0 mM HAuCl_4_·3H_2_O	nanoparticle	*E. coli*, *E. faecium*, *S. enterica*, *S. typhimurium*	In vitro	antibacterial, promoting cell proliferation, antioxidation	[36]
		2 wt% HAuCl_4_·3H_2_O	nanoparticle	*E. coli*, *S. aureus*	In vitro, rat model	antibacterial, promoting cell migration and proliferation	[75]
	ZnO	61.7 mM ZnONPs	film	*E. coli*, *S. aureus*	In vitro	antibacterial	[31]
		0.01 M ZnONPs	film	*E. coli*, *S. aureus*	In vitro	antibacterial	[76]
		24.7 mM ZnONPs	sponge	*E. coli*, *S. aureus*	In vitro	antibacterial	[32]
Natural extracts	plant extract	10 mg/mL lupeol	hydrogel	*E. coli*, *S. aureus*	In vitro, rat model	antibacterial, promoting cell migration and proliferation, anti-inflammatory	[64]
		3 mg/mL berberine	hydrogel	*E. coli*, *S. aureus*	In vitro	antibacterial	[35]
		200 mg VDMC/VBDMC	film	*A. baumannii*, *S. epidermidis*	In vitro	antibacterial, antioxidation, promoting cell migration	[62]
		3% turmeric extract	sponge	*E. coli*, *S. aureus*	In vitro	antibacterial, antioxidation, anti-inflammatory	[60]
	animal extract	2% *w*/*v* chitosan	hydrogel	*E. coli*, *S. aureus*	In vitro	antibacterial, antioxidation, promoting cell migration and proliferation	[77]
		2% *w*/*v* chitosan	scaffold	*E. coli*, *S. aureus*	In vitro	antibacterial	[61]
		0.4 mg/mL composite nanofiber solution	nanofibers	*E.coli*, *B. subtilis*	In vitro	antibacterial, promoting cell proliferation	[78]
		2% *w*/*v* chitosan	nanofibers	*E. coli*, *S. aureus*	In vitro	antibacterial	[63]
		20–75 mg/mL lysozyme	gel	*E. coli*, *S. aureus*	In vitro	antibacterial	[79]
Antibiotics		10 mg/mL gentamicin sulfate 30 mg/mL aspirin	hydrogel	*E. coli*, *S. aureus*, *P. aeruginosa*	In vitro	antibacterial	[28]
		vancomycin gentamicin	hydrogel	*MRSA*, *P. aeruginosa*, *E. coli*	In vitro, rat model	antibacterial, promoting angiogenesis, collagen deposition	[80]
		10% moxifloxacin	film	*A. baumannii*, *S. epidermidis*, *MRSA*	In vitro, rat model	antibacterial, promoting angiogenesis, collagen deposition	[65]
		32 μg/mL azithromycin	scaffold	*S. aureus*, *P. aeruginosa*, *E. coli*, *C. albicans*	In vitro, rat model	antibacterial, promoting cell migration and proliferation, promote angiogenesis, collagen deposition	[34]
		5 wt% tetracycline	nanofibers	*E. coli*, *S. aureus*	In vitro, rat model	antibacterial, promote angiogenesis, collagen deposition, anti-inflammatory	[81]
		0.015 g tigecycline	nanofibers	*E. coli*, *B. subtilis*	In vitro, rat model	antibacterial	[82]
		10 mg/mL Amoxicillin	nanoparticle	*S. aureus*, *K. pneumonia*, *E. coli*, *A. baumannii*, *P. aeruginosa*	In vitro, rat model	antibacterial, promote angiogenesis, collagen deposition	[83]
